# Exosomes as Smart Nanoplatforms for Diagnosis and Therapy of Cancer

**DOI:** 10.3389/fonc.2021.743189

**Published:** 2021-08-26

**Authors:** Yuying Zhao, Piaoxue Liu, Hanxu Tan, Xiaojia Chen, Qi Wang, Tongkai Chen

**Affiliations:** ^1^Science and Technology Innovation Center, Guangzhou University of Chinese Medicine, Guangzhou, China; ^2^Department of Mammary Disease, Guangdong Provincial Hospital of Chinese Medicine, The Second Clinical Collage of Guangzhou University of Chinese Medicine, Guangzhou, China; ^3^School of Fundamental Medical Science, Guangzhou University of Chinese Medicine, Guangzhou, China; ^4^State Key Laboratory of Quality Research in Chinese Medicine, Institute of Chinese Medical Sciences, University of Macau, Macau, China

**Keywords:** exosomes, biomarker, cancer diagnosis, cancer therapy, brain cancer

## Abstract

Exosomes are composed of a lipid bilayer membrane, containing proteins, nucleic acids, DNA, RNA, etc., derived from donor cells. They have a size range of approximately 30-150 nm. The intrinsic characteristics of exosomes, including efficient cellular uptake, low immunogenicity, low toxicity, intrinsic ability to traverse biological barriers, and inherent targeting ability, facilitate their application to the drug delivery system. Here, we review the generation, uptake, separation, and purification methods of exosomes, focusing on their application as carriers in tumor diagnosis and treatment, especially in brain tumors, as well as the patent applications of exosomes in recent years.

**Graphical Abstract:**
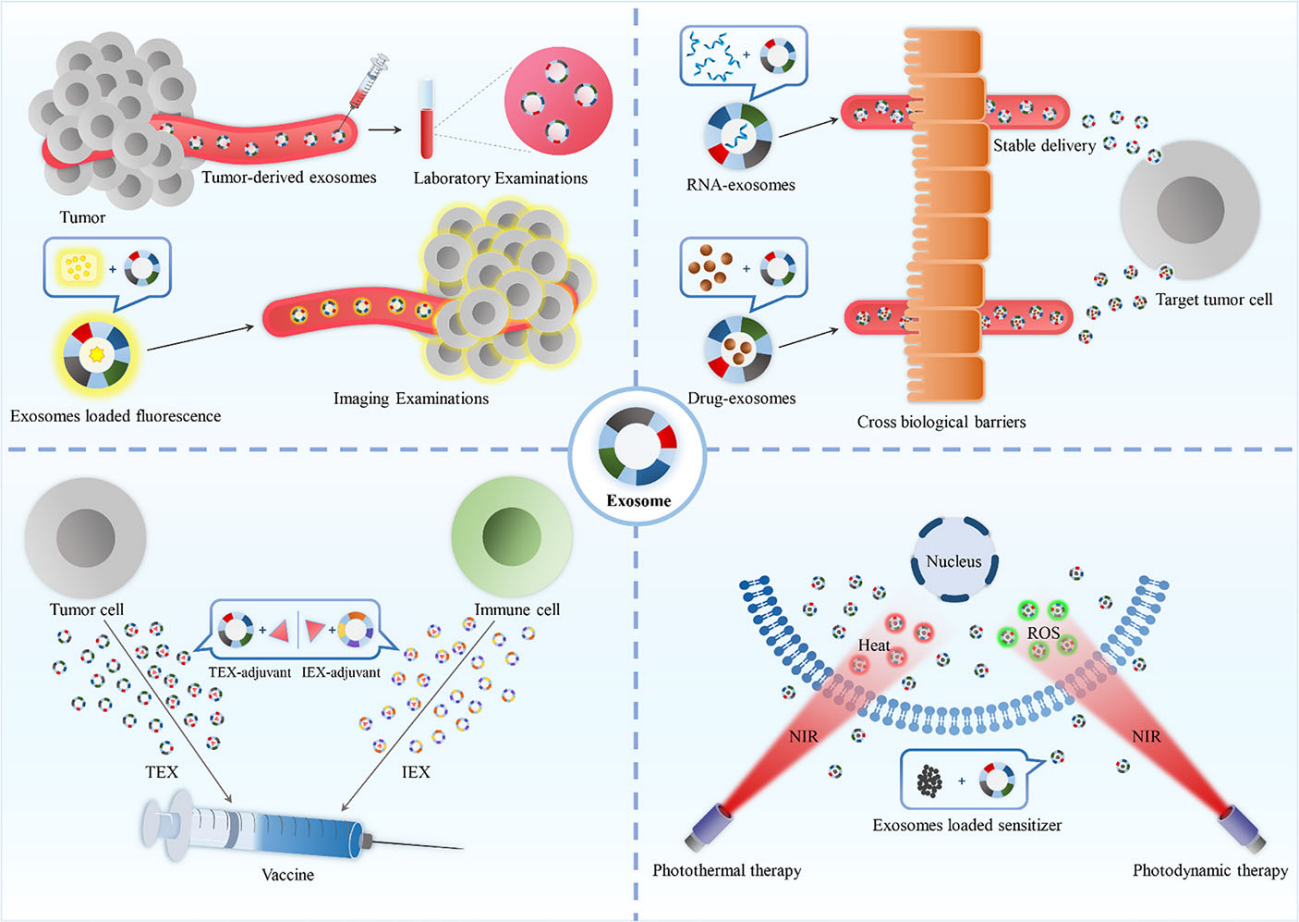


## Introduction

Most cells secrete various types of membrane vesicles, called extracellular vehicles (EVs) (1). EVs are classified into (1) ectosomes and (2) exosomes based on their size, biogenesis, and biophysical properties (2). Ectosomes are ubiquitous vesicles, approximately 100 to 500 mm in diameter, that are directly released from the plasma membrane (PM).

In the early 1980s, Pan and Johnstone first identified exosomes while studying the process of maturation of reticulocytes into erythrocytes (3). Exosomes are formed by the exocytosis of intraluminal vesicles (ILVs)-containing multivesicular bodies (MVBs) that are formed by the sequential invagination of the PM. The released exosomes have an approximate diameter of 40 to 160 nm (average ~ 100 nm) (4).

Exosomes possess a unique ability to facilitate complex biological responses, which promotes their use as diagnostic and therapeutic tools to treat various diseases, including neurodegenerative, cardiovascular, and cancer (5). The heterogeneity of exosomes is based on the different combinations of various characteristics, including size, original cells (source), content (cargo molecules), and effect on the functioning of the recipient cells (6). Recent studies are actively exploring exosomes as therapeutic agents to deliver drug payloads based on their ability to deliver various cargo molecules, including specific proteins, metabolites, lipids, and nucleic acids (7). Unlike liposomes, injected exosomes can effectively enter other cells and provide functional cargo molecules without getting attacked by the immune system and inducing toxicity due to its biogenetic derivation (8, 9).

Compared with other nanomaterials such as polymer-based nanomaterials and inorganic nanomaterials, they both have excellent pharmacokinetic properties, bioavailability (10), biodistribution, and can decrease the side effects of free drugs (11, 12). As a novel drug carrier, exosomes possess several unique structural and physicochemical properties, including low toxicity and immunogenicity, efficient cellular entry, intrinsic ability to cross biological barriers, increased circulatory stability, and active targeting potential (9). Interestingly, cancer cells are known to secrete excessive amounts of exosomes compared to normal cells and support tumor progression by promoting angiogenesis, modulating the immune system, and remodeling the surrounding parenchymal tissue (13, 14). Currently, there are several clinical trials with varying objectives, investigating the role of exosomes as tumor markers. This review summarizes the recent research on exosome as a carrier in tumor diagnosis and treatment, especially brain tumors, to facilitate the application of exosomes in the development of tumor therapy.

## Background of Exosomes

### Different Periods of Exosomes

#### The Biogenesis of Exosomes

**Figure 1:**
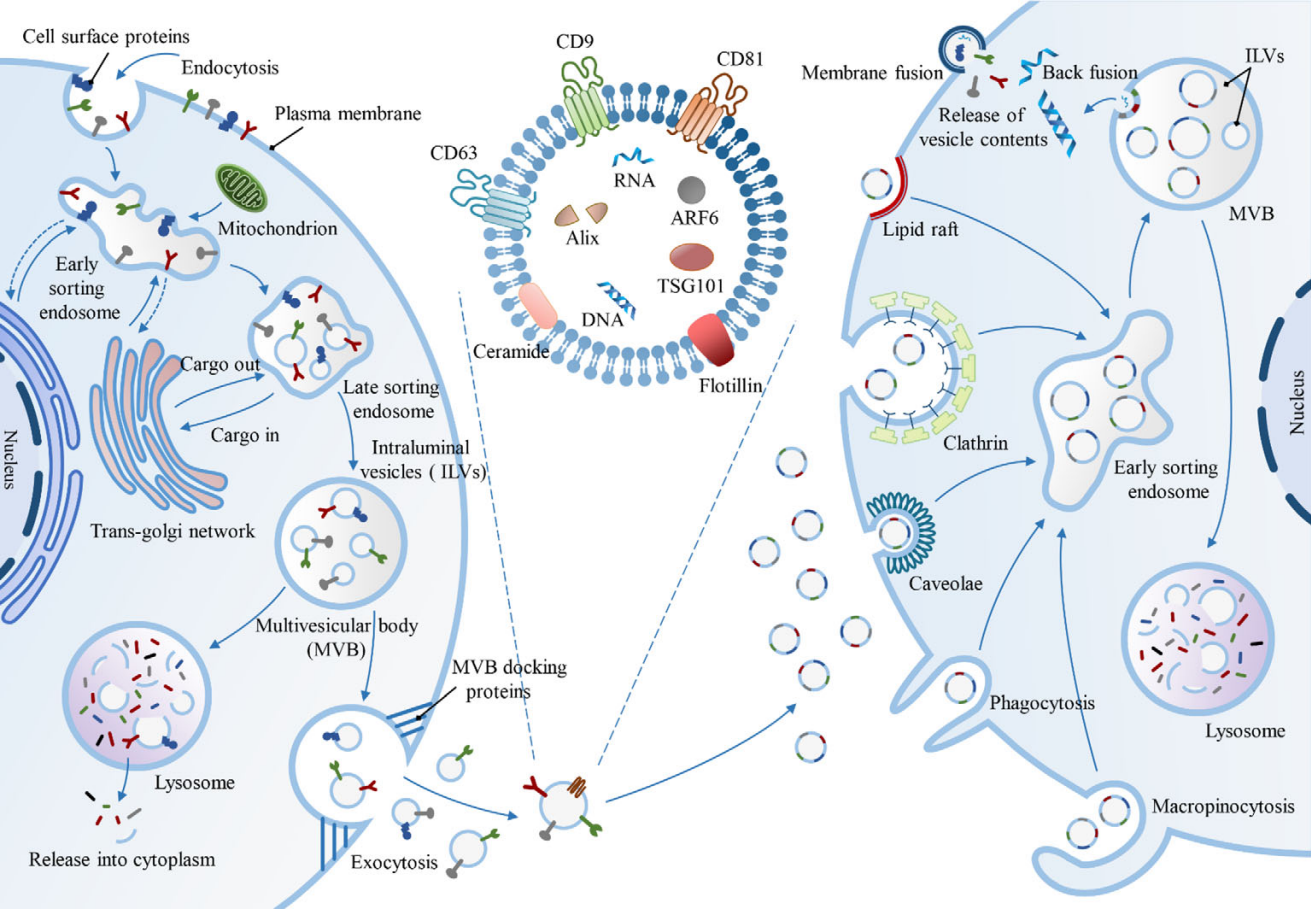
Biogenesis, biomarkers and uptake of exosomes.

Exosomes are derived from the early-sorting endosomes (ESEs), late-classified endosomes (LSEs), and ultimately ILVs-containing intracellular MVBs *via* the endosomal pathway (**Figure 1**). ESEs are cup-shaped structures formed by the initial invagination of the PM, which includes cell-surface proteins and extracellular soluble proteins. In some cases, the newly formed ESE might merge with the preexisting ESE. Consequently, ESEs mature into LSEs, leading to the formation of ILVs-filled MVBs *via* the second invagination of the PM. Ultimately, the fusion of MVBs with the PM results in the secretion of ILVs *via* exocytosis (15).

There are two major pathways involved in the generations of exosomes: Endosomal Sorting Complex Required for Transport (ESCRT)-dependent pathway and ESCRT-independent pathway (16). The ESCRT mechanism involves four major complexes, ESCRT-0, ESCRT-I, ESCRT-II, and ESCRT-III, comprising several subunits which play distinct roles. The ESCRT-0 complex identifies and separates ubiquitinated transmembrane proteins in the endosomal membrane; ESCRT- I, along with ESCRT-II, initiate the local budding of the endosomal membrane, followed by ESCRT-III-mediated vesicle segregation (17). The ESCRT-independent mechanism of exosome biogenesis requires the generation of ceramide by type 2-neutral sphingomyelinase (18). Additionally, ESCRT-independent endosomal sorting is regulated by proteins from the tetraspanin family and exosome-rich CD63 (19). Thus, it seems that both mechanisms might operate synergistically for the biogenesis of exosomes. Moreover, the cell type, cellular homeostasis, and cargo molecules could be vital for regulating the secretion of exosomes (20).

#### The Release (Secretion) of Exosomes

Exosomes are secreted as ILVs post the fusion of MVBs with the PM. Alternatively, MVBs are directed to lysosomes for degradation (18). The mechanism of regulation of the balance between intracellular degradation or the release of exosomes is unclear. Recent studies have provided insights into a possible mechanism, where the ISGylation of MVB proteins facilitates their fusion with lysosomes, directing MVBs toward the degradation pathway and away from the secretory pathway (21); intracellular transport of MVB *via* microtubules to the PM (22), the formation of docking sites in the PM (23), or recruitment of Soluble NSF Attachment Protein Receptor (SNARE) proteins and synaptotagmin family members that facilitate their fusion with either lysosomes or the PM (24). Molecular regulators associated with the release of exosomes include several molecules involved in MVB docking, such as RAB27A and RAB27B32 and their respective effectors, synaptotagmin-like protein 4 and exophilin 5 (20). Furthermore, studies have shown that autophagosomes fuse with MVBs and direct them to lysosomes to prevent exosome secretion (20). Similarly, previous studies have been shown that the prion protein (PrP) inhibits the formation of autophagosomes by interacting with caveolin to promote exosome secretion (25).

#### The Uptake of Exosomes

There are several pathways involved in the uptake of exosomes, including receptor-mediated endocytosis, clathrin-mediated endocytosis, lipid raft-mediated endocytosis, phagocytosis, caveolin-mediated endocytosis, micropinocytosis (26), and membrane fusion (27), which increase the complexity of exosomes in intercellular communication (**Figure 1**). For instance, mutated KRAS expression generates oncogenic signals that promote the uptake of exosomes in human pancreatic cancer cells through macrocytosis (28), clathrin-dependent endocytosis mediates exosome uptake in neurosecretory PC12 cells (derived from rat adrenal medullary tumor) (29).

A recent study identified a different exosome internalization pathway involving filopodia-mediated recruitment, followed by endocytosis by a process resembling virus entry (30). However, variable conditions impact exosome uptake and subsequent biological activity, such as the preferential uptake of small exosomes by cells of different sizes, exosome uptake is inhibited at low temperatures (31). Some proteins are known to facilitate exosome uptake, such as the tetraspanin membrane proteins CD9 and CD81 and intercellular adhesion molecule (ICAM)-1 (32). Additionally, proton pump inhibition or cellular pH changes in melanoma cells are known to limit exosome uptake (33).

### Content and Function of Exosomes

Previous studies on the composition of exosomes have revealed the presence of various cargo molecules, including membrane proteins, cytosolic and nuclear proteins, extracellular matrix proteins, metabolites, and nucleic acids, such as mRNA, noncoding RNA species, and DNA (34, 35). The composition can vary widely based on cellular origin, heterogeneity in size, cellular microenvironment, and inherent biology of cells (4).

The diverse composition of exosomes results in the complexity of their functions. Additionally, the effects of exosomes on receptor cells vary based on the expression of receptors on the cell surface. Generally, exosomes are involved in eliminating excess metabolites to maintain cellular homeostasis (36) and intercellular communication, which has been associated with numerous physiological and pathological functions (37). In cancer cells, exosomes are known to support tumor progression by promoting angiogenesis, regulating the immune system, and remodeling the surrounding parenchymal tissue (38). In breast milk, breast milk-derived exosomes contain miRNAs with immunomodulatory functions to regulate immune tolerance and promote postnatal health and growth (39).

Recent studies on the central nervous system have indicated that exosomes secreted by oligodendrocytes enhance neuronal viability as well as an increase in the neuron firing rate (40). In a syngeneic mouse model of melanoma, melanoma-derived exosomes induced lung vascular leakiness and recruited bone marrow-derived macrophages to metastatic sites. Moreover, chemotherapy and radiation therapy also impact the composition and functioning of exosomes with potential implications on therapy outcomes (41).

### Separation and Purification of Exosomes

Current studies are involved in the exploration of exosomes as a new method for the cellular delivery of drug payload. There are several techniques being used to separate exosomes from EVs (**Table 1**); however, there are still many challenges regarding the efficient and economic production, separation, and purification of exosomes in adequate quantities, which prevent their clinical use (9).

**Table 1:** Isolation technique of exosomes.

Isolation technique	Isolation methods	Advantages	Limitations	Ref.
Ultracentrifugation	Differential centrifugal forces	Low equipment cost, fast procedure and high yield	Time-consuming, costly instrumentation; exosomes may be damaged by repeated centrifugation	(42, 43)
Sucrose-gradient centrifugation	Ultracentrifugation combined with sucrose density gradients	Yields highly pure exosomes.	It is difficult to achieve pure vesicle purification through density-based procedures, which might provide enrichment at best.	(44)
Size-exclusion chromatography	Separation based on gel filtration	The biological activity and integrity of the isolated exosomes are retained.	Since it is a time-intensive process, the processing of several samples is difficult.	(45)
Immunoaffinity capture	Positive selection: magnetic bead-bound antibodies bind to targeted exosomes. Negative selection: magnetic bead-bound antibodies bind and isolate irrelevant exosomes.	Highly efficient, can be used for quantification and qualitative analysis of the isolated exosomes.	Expensive; the strong antibody-antigen binding makes the dissociation of bound exosomes difficult for further analyses.	(46)
Polymer-based precipitation	Polymers, such as PEG, considerably reduce the solubility of exosomes, which can then be isolated *via* low-speed centrifugation.	Easy operation, rapid processing, does not require specialized equipment.	Expensive; exosome isolation may contain contaminating proteins.	(47, 48)
Microfluidics-based isolation	This microscale technique uses differences in size, density, and immunoaffinity to isolate exosomes.	Rapid, great precision, and sensitive detection	Low yield	(49, 50)

Therefore, it is imperative to promote a standardized method that is practical, efficient, and feasible to isolate and purify exosomes from the cell culture medium.

## Applying of Exosome to Improve Cancer Diagnosis

Timely detection is vital for the effective treatment of cancer. Currently, clinical diagnosis of cancer includes laboratory examinations, imaging studies, and tissue biopsy which is regarded as the gold standard. However, since biopsies are invasive, the first two methodologies are more widely used. Unfortunately, laboratory examination and imaging analysis have low sensitivity and have an insignificant role in the diagnosis of early-stage cancer. For several years now, scientists have discussed the application of exosomes in clinical diagnosis, especially in improving the sensitivity of laboratory and imaging studies. Moreover, an early cancer diagnosis is directly related to improved survival rates.

### Application in Laboratory Examinations

Exosomes can transport DNA, RNA, and proteins into biological fluids to mediate intercellular communication (4). Tumor cell-derived exosomes contain oncogenic information that facilitates cancer growth and metastasis (51). Thus, it was recently predicted that exosomes could act as tumor biomarkers (52, 53). A study showed that TAG72-rich and CA125-rich exosomes could act as biomarkers of colorectal cancer (54). A significant increase in the serum/plasma levels of miR-547-3p was observed in patients with osteosarcoma (55) or hepatocellular carcinoma (56) and considered as a diagnostic biomarker, and miR-547-3p levels were significantly reduced post-radiotherapy in patients with glioma (57). Exosomes can be detected in the early stages of cancer, suggesting that they might be vital candidates for laboratory examinations.

**Figure 2:**
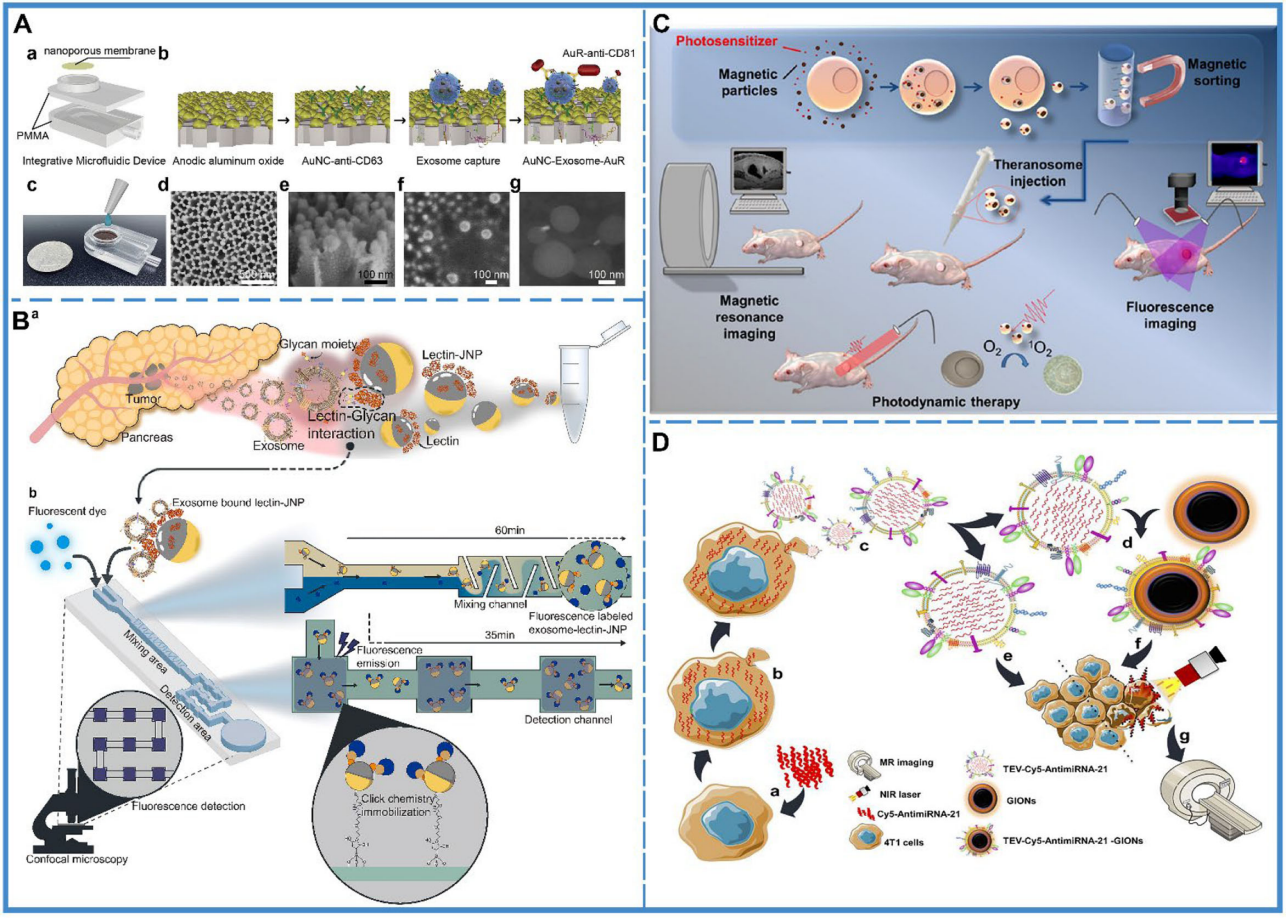
Cancer diagnosis. **(A)** An integrative microfluidic device (IMD) for the isolation and detection of exosomes. (a) The model design of the IMD. (b) The *in-situ* detection of exosomes. (c) An image of the IMD. (d) A scanning electron micrograph of the Au nanoparticles deposited on a 50-mm-thick AAO membrane; side view presented in (e). (f) A scanning electron micrograph of the captured exosomes on the AAO membrane, and (g) A scanning electron micrograph of the AuNC-Exosome-AuR complex. Reproduced with permission from Ref (60). Copyright 2020 Elsevier. **(B)** Schematic illustration of the capturing of pancreatic cancer exosomes *via* Exo-chip system. (a) Isolation of the exosomes from the patient blood specimens/pancreatic cancer cell line culture medium. (b) After addition to the Exo-chip with Janus nanoparticles (JNPs), the sample was visualized *via* fluorescence imaging and analysis. Lectin-conjugated JNPs mediate the interaction between exosomes and the chip substrate *via* click reactions (maleimide-functionalized JNPs and thiol-functionalized substrate) that bind the substrate in a laminar flow system. Reproduced with permission from ref (58). Copyright 2020 Elsevier. **(C)** Schematic illustration of the synthesis of tumor derived extracellular vehicles loaded Cy5 and antimiRNA-21 (TEVs-Cy5-antimiRNA-21) and TEVs-Cy5-antimiRNA-21-coated gold iron oxide nanotheranostics (GIONs). (a) Cy5-antimiRNA-21 was transfected into the donor 4T1 cells, (b) Cy5-antimiRNA-21-packed donor 4T1 cells, (c) *In situ* generation of 4T1 tumor cell-derived Cy5-antimiRNA-21-loaded extracellular vesicles, (d) Top-down fabrication synthesis of TEVs-Cy5-antimiRNA-21-coated GIONs, (e) TEVs-Cy5-antimiRNA-21-mediated cancer therapy, (f) TEVs-Cy5-antimiRNA-21-GIONs-mediated multimodal imaging and photothermal cancer therapy, (g) MRI of 4T1 cancer cells delivered by TEVs-Cy5-antimiRNA-21-GIONs. Reproduced with permission from ref (59). Copyright 2013 American Chemical Society. **(D)** Schematic representation of theranosome generation from drug-loaded magnetic precursor cells along with their application in photodynamic therapy and dual-mode MRI and fluorescence imaging. Reproduced with permission from ref (66). Copyright 2018 American Chemical Society.

Tumor-derived exosomes possess tumor markers, and tumors can be identified based on the levels of tumor-derived exosomes in bodily fluids (**Figure 2A**) (60). However, during the early stages of development, tumors secrete few exosomes, which are difficult to detect, and thus, detachable microfluidic devices were invented to detect early-stage tumors using electrochemical aptamer sensors (DeMEA). This equipment can improve the sensitivity of the detection of tumor-secreted exosomes (61). It provides the possibility of early detection of cancer. When a tumor progresses to a terminal stage, it is critical to determine its level of metastasis to develop the treatment strategy. Thus, an ingenious microfluidic device was designed to detect exosomes based on the specific binding of lectins to unique glycans on the surface of cancer cell-derived exosomes, which could distinguish between metastatic and non-metastatic pancreatic cancer while improving the accuracy of pancreatic cancer diagnosis (**Figure 2B**) (58).

### Application in Imaging Examinations

Compared with laboratory examinations, imaging examinations are more intuitive and visual (62). Exosomes are considered to be natural delivery vesicles that can slow down the endocytosis of macrophages and extend blood circulation time to avoid clearance *via* the kidney, liver, and the immune system (28), implying that their biocompatibility and transcellular permeability present them as probable vehicles for theranostic applications. Currently, for imaging analysis, the cargo molecules that can be loaded by the exosomes include inorganic molecules (63), magnetic materials (**Figure 2C**) (59), and fluorescent molecules (64). Besides, there is two main preparation method of exosome theranostics: *in-vitro* biosynthesis and in-situ biosynthesis.

*In-vitro* biosynthesis is a more controllable technique. In a study, chlorin e6 (Ce6) molecules were loaded on the surface of inorganic Au nanoparticles (Au@Ce6), followed by embedding the Au@Ce6 into exosomes, which were collected and purified from the urine of gastric cancer patients. The results showed that the nanovehicles could successfully improve the cellular uptake of cancer cells and reduce the endocytosis of macrophages, which further enhanced the accumulation of targeted tumor sites for superior real-time fluorescence imaging (63). Another study used superparamagnetic iron oxide (SPIO)-loaded exosomes for magnetic particle imaging (MPI), which enabled the acquisition of *in vivo* images with greater contrast, sensitivity, and quantitation than magnetic resonance imaging (MRI) (65). Apart from this, gold-iron oxide nanoparticles-loaded exosomes also demonstrated excellent T2 contrast in MRI (**Figure 2D**) (66).

*In-situ* biosynthesis is a more sensitive technique where the raw materials of the bioprobe are injected into the organism, followed by their self-assembly in the tumor microenvironment (TME). After the bioprobe assembly is complete, it gets embedded into the exosome as metabolic trash and excreted outside the cell. Thus, it can be found in the early stages of cancer. A study utilized TME for bioimaging with in-situ biosynthesized nanoscale gold and iron probes and subsequently disseminated Au-Fe nanoclusters from cargo exosomes within the circulation, which could be subsequently used as fluorescence, Computed Tomography (CT), and MRI imaging tools for cancer diagnosis (67).

### Application in Brain Cancer Diagnosis

In clinical studies, the application of biopsy to brain cancers is limited due to the location of the nidus. Usually, the diagnosis is confirmed by imaging analysis and laboratory examination, and biopsy is performed only when there are clinical symptoms; however, by then, the cancer has developed to middle or advanced stages. Therefore, it is necessary to improve the sensitivity of imaging techniques and laboratory examination and strive for early diagnosis. The use of exosomes in the diagnosis may solve this problem. In the laboratory diagnosis, once the TME is established, exosomes containing oncogenic information are released into the peripheral blood, which can be detected by laboratory tests (57). As for the image-based diagnosis, encapsulating particles with imaging capabilities in exosomes can be absorbed by the brain cancer cells due to their capability to cross the blood-brain barrier (BBB) (65, 68) or be transported from the nidus to the peripheral blood (67), which can effectively improve the imaging signals of CT or MRI and enhance the sensitivity of imaging diagnosis. Based on these conclusions, the combination of improved laboratory tests and imaging techniques by exosome can facilitate early diagnosis of brain cancers, reduce the rate of missed diagnosis, and achieve early treatment of brain cancers.

In summary, exosomes could improve the sensitivity of laboratory tests and imaging to detect tumors. In laboratory examinations, the presence of exosomes with tumor markers in bodily fluids could be used to detect tumors. Further research invented numerous devices to improve the sensitivity of detecting early tumors as well as for distinguishing whether tumors were metastatic by analyzing exosomes, with improved clinical efficacy. In imaging examinations, exosomes derived from tumor cells were found to enhance the signal at the tumor site by loading inorganic molecules, magnetic materials or fluorescent molecules. These involved two methods: (1) *in vitro* biosynthesis where fluorescent molecules were loaded into the exosomes *in vitro* and then transported to tumors through the homing ability of exosomes; (2) in-situ biosynthesis where the raw materials of the bioprobe were injected into the organisms and then self-assembled in the tumor microenvironment (TME).

## Use of Exosomes to Improve RNAi-Based Cancer Therapy

**Figure 3:**
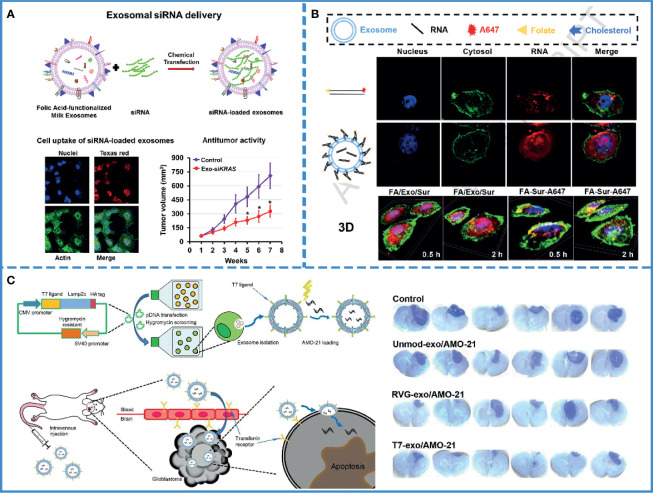
RNAi-based cancer therapy. **(A)** Schematic illustration of folic acid-functionalized milk exosomes loaded with siRNA *via* chemical transfection. Reproduced with permission from ref (69). Copyright 2019 Elsevier. **(B)** FA-decorated exosomes loaded with siRNA were uniformly distributed in the cytoplasm, and there was little overlap with the endosomes/lysosomes in the cells, indicating that exosomes with folate targeting had effective cytosol delivery property. Reproduced with permission from ref (100). Copyright 2019 Elsevier. **(C)** Schematic illustration of the synthesis of a T7 peptide-decorated exosome (T7-exo). After systemic administration, the T7-exo could cross the BBB, bind to glioblastoma, and enhance the efficiency of AMO delivery. Reproduced with permission from ref (79). Copyright 2019 Elsevier.

Until now, RNAs interference (RNAi)-based therapy, such as small interfering RNAs (siRNAs) and micro RNAs (miRNAs) (**Table 2**), have been demonstrated to be effective for cancer treatment (85, 86). However, rapid hydrolysis, poor bioavailability (87), and the inability to cross the biological barriers have restricted the development of RNAi-based therapies (88). Thus, an effective delivery system is needed that protects RNAs from nuclease degradation and releases them into the cytoplasm of targeted cells without adverse effects (89). Currently, there exist three classes of delivery vehicles: viruses, polycationic polyethyleneimine (PEI)-based nanoparticles, and liposomes. With the delivery of viruses, RNAs can enter host cells and survive in host cells for a long time *via* genome integration. But there are some serious drawbacks to using viruses. They get cleared by preexisting antibodies in the bloodstream and active complement or coagulation factors in the circulating blood; furthermore, gamma retroviral vectors were used for clinical gene therapy in ten patients with X-linked SCID (SCID-X1), and four of them developed T-cell leukemia after treatment. Viruses may also cause malignancies (90). PEI nanoparticles or liposomes, which are more safe compared with viruses, can protect RNAs from degradation in the bloodstream and facilitate cellular uptake, but they may induce cellular stress, inflammatory response, and apoptosis (91). On the contrary, exosomes have high target specificity, high permeability, and low toxicity. Meanwhile, they also provide serum stability (**Figure 3A**) (69). and facilitate cellular uptake by cancer cells, without immune response or inflammatory reaction (4). Given the limitations of these three vehicles, exosomes, a natural intercellular communication system, are more suitable vehicles over existing RNAs delivery vehicles (93, 94).

**Table 2:** Exosome-mediated siRNA delivery.

Tumor	Source of exosomes	Cargos	Loading methods	Drug loading (%)	Encapsulation efficiency (%)	Zeta potential (mV)	Particle size (nm)	Improvements	Ref
Lung cancer	Bovine milk	siRNA	Electroporation and chemical transfection (Exo-Fect) methods.	30	/	/	/	More stale, higher drug delivery efficiency, higher cellular uptake.	(69)
Lung cancer	tLyp-1-lamp2b transfected HEK293T cell	siRNA	Electroporation	/	61.53 ± 0.32	/	~100	Desirable targeting efficacy.	(70)
Bladder cancer	Bone marrow derived mesenchymal stem cells	microRNA-9-3p	/	/	/	/	~120 nm	Desirable inhibition of tumor growth and metastasis.	(71)
Human Prostate Cancer	Human bone marrow-derived MSC (BMSC)	microRNA-143	/	/	/	/	132.5 ± 37.4	Desirable targeting efficacy.	(72)
Pancreatic ductal adenocarcinoma	Human umbilical cord mesenchymal stromal cells	miR-145-5p	Chemical transfection (Exo-Fect)	/	/	/	119	Higher delivery efficiency.	(73)
Gastric cancer	HEK293T cells	miR-374a-5p inhibitor	Chemical transfection (Exo-Fect)	/	/	/	/	More stale, higher drug delivery efficiency, lower toxicity and immunogenicity.	(74)
Oral squamous cell carcinoma	T cells	miR-138	Chemical transfection (Exo-Fect)	/	/	/	50 - 200	Desirable targeting efficacy, lower toxicity.	(75)
Malignant mesothelioma	HUVECs	MiR-126	Chemical transfection (Exo-Fect)	/	/	/	/	Desirable therapeutic efficacy to cancer stroma.	(76)
Hepatocellular carcinoma	Plasma	miR-31 and miR-451a	Middle electroporation	/	31.63 ± 5.94	/	/	Less enzymatic degradation of miRNA, desirable targeting efficacy.	(77)
Pancreatic Cancer	Bone Marrow Mesenchymal Stem Cell	MicroRNA-126-3p	/	/	/	/	100	Desirable targeting efficacy.	(78)
Glioblastoma	293T cell	microRNA-21	Electroporation	1.68 ± 0.23	/	-10 and -3	15 - 50	Higher BBB transportation and delivery efficiency of AMO	(79)
Colorectal cancer	THLG-293T or LG-293T cells	miR-21 inhibitor	Electroporation	5-FU: 3.1 miR-21i: 0.5	/	− 11 ± 2.7	110 ± 11.3	Higher cellular uptake, desirable targeting efficacy.	(80)
Breast Cancer	Mesenchymal Stem Cells	LNA-antimiR-142-3p	Electroporation	/	57	/	103	Desirable ability to penetrate the cancer stem cells	(81)
Myeloid leukemia	Human bone marrow mesenchymal stem cells	miR-222-3p	chemical transfection (Exo-Fect)	/	/	/	~200	Desirable targeting efficacy.	(82)
Breast cancer	Breast cancer cells	siRNA (siS100A4)	incubation and extrusion method	/	86.70 ± 1.22	-33.61 ± 0.81	173.63 ± 0.77	Less degradation in blood circulation, desirable inhibition of tumor growth and metastasis.	(83)
Digestive system tumors	Milk	bcl-2 siRNA	Ultrasound method	66.9 ± 4.5	/	/	60 - 90	Higher cell membrane crossing efficacy and less degradation in serum.	(84)

“/” represents that information was not mentioned in the original article.

### Application in Stable Delivery

As mentioned above, the use of RNAs in oncology has been limited by their easy degradation by nucleases, which reduces their stability in the blood (95). Furthermore, the pore size of the glomerular basement membrane (GBM) is approximately 6-10 nm (96), while the length of naked RNA is only 7 nm; thus, it can be filtered through GBM and pass into the urine quickly (97). Currently, scientists assume that this defect in RNAs could be remedied by using the exosome. Several experiments have been designed to verify this hypothesis. For example, one study found a significant increase in the storage stability of RNAs after loading with exosomes compared with only PEIs-modified siRNAs (92). Plasma-derived exosomes also protected miRNAs from nuclease degradation and successfully promoted apoptosis of the HepG2 hepatocellular carcinoma cells (77). Again, siRNAs loaded with milk-derived exosomes are stable and resist degradation; the combination of exosomes and siRNAs eventually silence the expression of target genes by approximately 2-10 folds (**Figure 3A**) (69). Thus, exosomes, with no inherent toxicity and intrinsic biocompatibility, can protect RNAs from nuclease degradation and renal filtration and help maintain the stability of RNAs in blood.

### Application in Crossing Biological Barriers

However, only maintaining stability is not enough. Since naked RNAs have a poor ability of cell penetration, it is hard for RNAs to enter into the cell (98). Even after entering the cell, they run the risk of being hydrolyzed by lysosomes (89). Consequently, the success of therapeutic RNAs depends on efficient cell internalization and timely endosomal escape. It is well known that exosomes have the innate ability to cross biological barriers; thus, engineered exosomes could efficiently facilitate cellular uptake of miRNAs and significantly reduce cancer proliferation (80). Professor Lin has proved that milk-derived exosomes can transport across the gastrointestinal barrier (99), and small interfering RAN (bcl-2 siRNA) encapsulated by milk-derived exosomes can effectively pass through cell membranes and inhibit the growth of cancer cells (84). Additionally, a study demonstrated that sometimes, exosomes fused with the target cell membranes, releasing their cargos directly into the cytoplasm. Thus, exosomes could help avoid the endosome trapping, enabling the full functionality of the RNAs and effectively inhibiting tumor growth (**Figure 3B**) (100). If the exosome was internalized *via* the clathrin-mediated or clathrin-independent endocytosis, it could also help RNAs escape digestion by reverse fusion with the limiting membrane of the MVE or by being re-secretion *via* the early endocytic recycling pathway (30).

### Application in Overcoming Off-Target Effect

RNA therapy runs the risk of off-target effects, which can result in unanticipated phenotypes (89). Currently, active targeting might constitute the best way to solve this problem. Compared with other vehicles, exosomes have better targeting ability. First, exosomes have been proved to have a weak homing effect, i.e., cancer-derived exosomes are preferentially absorbed by the cancer cells (65, 101). Second, the exosomes originate from the cell membrane (4); thus, they can be modified *via* different targeted peptides or proteins and then can actively target cancer cells. Recently, exosomes were modified by a variety of target ligands, such as tLyp-1 (70), folic acid (69), iRGD peptide (102), and T7-peptide (79) in RNAs-based cancer therapy. Thus, this approach can limit the off-target effects and promote cellular uptake of RNAs by cancer cells, either passive targeting or active targeting.

### Application in Brain Cancer RNAs Interference Based Therapy

The poor diagnostics, therapeutics, and prognosis of brain cancer are well known. It mainly includes glioblastoma, medulloblastoma, and oligodendroglioma. Currently, a standard treatment for brain cancer is surgical resection, followed by radiotherapy and chemotherapy. However, surgical resection is limited because of the diffusive nature of brain cancer and the intolerance in some patients. Also, chemotherapy and radiotherapy can have serious adverse effects; thus, brain cancer has a low cure rate and a high recurrence rate clinically. Therefore, a drug with low-toxicity, low-immunogenicity, high bioavailability, ability to cross the blood-brain barrier (BBB), and active targeting is needed. Most naked molecules do not meet these requirements. Fortunately, exosomes possess an invisibility cloak, increasing their functionality by wrapping them in a vesicle. In other words, they can carry the cargos and pass through biological barriers smoothly, including BBB; thus, improving the biological availability of cargos (103). Additionally, exosomes are non-toxic, which will not lead to acute immune rejection, thus giving the cargos high biocompatibility (7). Also, exosomes present a customizable surface and can be modified for different applications (104). These characteristics of exosomes are exclusive to them (105), making them the preferred carriers in brain treatment.

For the treatment of brain tumors, RNA therapy is not only hindered by the BBB but also by the off-target effects. The complex and mysterious structure of the brain makes the adverse effects of off-target unpredictable. One study analyzed the delivery of miRNA-21 using T7-peptide-decorated exosomes *via* intravenous injection. After systemic administration, this compound showed a brilliant brain-targeting ability and reduced tumor size in orthotopic glioblastoma models without adverse effects. Similarly, Lamp2b and rabies virus glycoprotein (RVG) are also known to target glioblastoma (**Figure 3C**) (79). If unmodified exosomes are used to deliver RNAs, then intra-tumor injection may be the most efficient and safe method (106).

In summary, exosome-loaded RNA for tumor treatment could improve the disadvantages of RNA, such as rapid hydrolysis, poor bioavailability, and inability to cross the biological barriers. Experiments have shown that exosomes can store RNA, avoiding RNA degradation by enzymes and maintaining its stability in the blood. In addition, exosomes can carry RNA across biological barriers, such as the blood-brain barrier and the gastrointestinal barrier. Finally, RNA therapy has the risk of off-target effects. Thus, exosomes can help RNA overcome off-target effects and improve targeting ability, in turn improving anti-tumor efficacy.

## Use of Exosome to Improve Cancer Chemotherapy

Several chemotherapeutic drugs, which exhibit potent anti-cancer effects *in vitro*, do not show similar efficacy *in vivo* due to low bioavailability and low biocompatibility. Also, long-term chemotherapy induces drug resistance in cancer cells (107), which causes further complications. A drug needs a drug delivery system to exert its therapeutic effect *in vivo* to improve its bioavailability, as well as its solubility in water, stability in blood, permeability to biological barriers, and accumulation in cancer tissues. Additionally, it is also necessary to improve their active targeting ability, hide their immunogenicity, and make them self-degradable. Exosomes as “natural nanoparticles,” can not only meet the above requirements but also help to overcome cancer drug resistance; thus, they can work as valuable carriers for drug delivery (**Table 3**) (118).

**Table 3:** Exosome-mediated anti-tumor drug delivery.

Source of exosomes	Drugs	Tumor	Drug loading (%)	Encapsulation efficiency (%)	Zeta potential (mV)	Particle size (nm)	Improvements	Ref
Bovine milk	Paclitaxel	Lung cancer	8	/	-7	75 ± 0.6	Improve oral bioavailability	(99)
Bovine milk	Curcumin	Cervical cancer	18–24	53.9 ± 6.7	/	93 ± 6	Improve low water solubility, rapid intestinal/hepatic metabolism, oral bioavailability	(108)
Bovine milk	Bilberry-derived anthos	Lung cancer	20	/	/	83 ± 1.7	Improve low solubility, low permeability, and poor oral bioavailability	(109)
FBS	Curcumin	Breast cancer	/	/	-24.1 ± 2.2	122.7 ± 6.5	Cross the BBB and facilitate accurate glioma recognition and improvement of the curative effect	(110)
Macrophage	Paclitaxel	Lung cancer	33	/	-4.4 ± 0.1	280.8 ± 3.1	High loading capacity, profound ability to accumulate in cancer cells, and improved therapeutic outcomes.	(111)
LIM1215 cells	Doxorubicin	Colorectal Cancer	2.60	9.06	-9.57 ± 0.38	187.83 ± 6.76	Improve the therapeutic effect of Dox and reduce its systemic toxicity.	(112)
MCF7, Caco2, PC3 and HepG2 cell lines	Bioactive compounds from black bean extract	Gastric cancer	Soyasaponin α: 17.31 soyasaponin β: 16.08	/	-19.23 ± 2.42	142.80 ± 23.67	Improve low solubility, poor penetration into cells, hepatic disposition, narrow therapeutic index, and rapid uptake by normal tissues	(113)
J774A.1 cells	Doxorubicin	Breast cancer	99	/	-26 ± 3	177 ± 21	Higher drug accumulation in target cells and improve small molecule stability and blood circulation time	(114)
Breast and colorectal cancer cells	Aspirin	Breast and colorectal cancer	/	/	/	50-150	Improve the poor water-solubility of aspirin	(115)
Bone marrow mesenchymal stem cells	Pancreatic ductal adenocarcinoma, paclitaxel (PTX) and gemcitabine monophosphate (GEMP)	Pancreatic cancer	GEMP: 8.78 PTX: 1.25	GEMP: 5.92 PTX: 2.62	-10.46 ± 0.55	75.5 ± 1.4	Surpass the restrictions of pathological ECM and increase the accumulation of therapeutics in the tumor site.	(116)
Autologous pancreatic cancer	Gemcitabine	Pancreatic cancer	11.68 ± 3.68	/	/	70 - 150	Facilitate cellular uptake of GEM and contributed to significantly increased cytotoxic effect of GEM	(117)

“/” represents that information was not mentioned in the original article.

### Use in Enhancing Bioavailability

Several natural products and extracts, such as paclitaxel, curcumin, celastrol, anthocyanin, etc. suffer from low bioavailability *in vivo* despite having excellent anti-cancer properties. Numerous experiments have shown that exosomes can improve the bioavailability of these drugs and improve their efficacy in cancer treatment. For example, aminoethylanisamide-polyethylene glycol-vectorized exosomes loaded with paclitaxel (AA-PEG-exoPTX) exhibit a high loading capacity, excellent accumulation in cancer cells upon systemic administration, and improved therapeutic efficiency (111). In this previous study, AA-PEG-vectorized exosomes increased the aqueous solubility of PTX and promoted the accumulation of PTX in tumor tissues. In another experiment, the poor aqueous solubility and instability in the blood of celastrol were improved *via* exosome wrapping. Thus, exosomes improved the bioavailability of celastrol and enhanced its anti-cancer efficacy (119).

Compared with systemic administration, oral administration is non-invasive and easy for the patient; thus, it constitutes an excellent treatment method for chemotherapy. However, the gastrointestinal barrier hinders the absorption of drugs, especially gastric acid, which reduces the stability of drugs. Fortunately, milk-derived exosomes can stably exist in gastric acid; thus, avoiding the degradation of the cargo molecules (99). Curcumin is well known for its excellent anti-cancer properties but poor aqueous solubility. Its hydrophobic nature results in not only poor aqueous solubility but also rapid intestinal hepatic metabolism. A study showed that exosome-encapsulated curcumin could stably exist in gastric acid and smoothly cross the gastrointestinal barrier to improve the bioavailability of curcumin. Additionally, compared with the pure curcumin group, exo-curcumin showed significantly improved accumulation in tumor cells as well as enhanced anti-tumor efficacy (108).

### Application in Enhancing Biocompatibility

**Figure 4:**
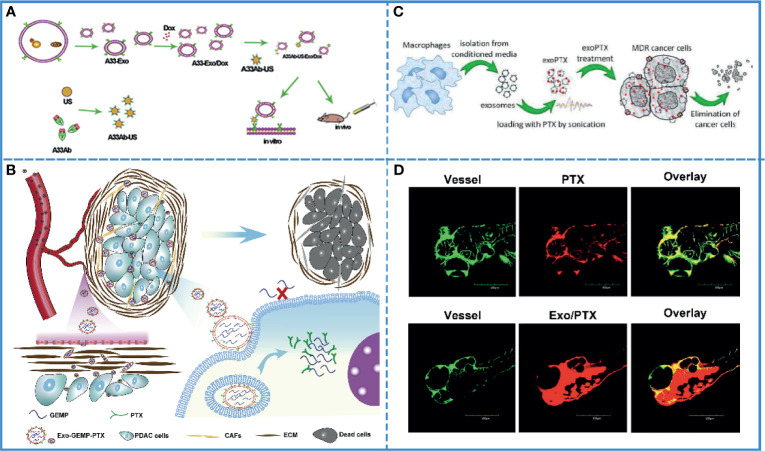
Cancer chemotherapy. **(A)** Schematic illustration of A33Ab-US-Exo/Dox complex formation between A33 antibody-coated surface-carboxyl Fe_3_O_4_ superparamagnetic nanoparticles (US) (A33Ab-US) and exosomes (A33-Exo) released from doxorubicin-loaded LIM1215 cells (A33-Exo/Dox). Reproduced with permission from ref (112). Copyright 2018 Elsevier. **(B)** Exosomes loaded with GEMP and PTX were used for targeted chemotherapy of pancreatic cancer *via* dual functions, i.e., stromal penetrability and anti-matrix and overcame chemoresistance. Reproduced with permission from ref (115). Copyright 2019 Elsevier. **(C)** Paclitaxel (PTX)-loaded exosomes (exoPTX), derived from autologous macrophages, exhibited high loading capacity, sustained drug release, profound ability to accumulate in resistant cancer cells, and elevated cytotoxicity compared to PTX. Reproduced with permission from ref (123). Copyright 2016 Elsevier. **(D)** Injection of fluorescence-labeled PTX alone and those complexed with bEND3 exosomes in zebrafish embryos showed that Exo/PTX effectively crossed the BBB into the brain. Reproduced with permission from ref (124). Copyright 2015 Springer Science.

Most chemical drugs, such as adriamycin, cisplatin, oxaliplatin, etc. suffer from low biocompatibility. The adverse effects of these drugs limit their clinical application. Theoretically, carriers with active targeting ability, low immunogenicity, and self-degradability should solve this problem. Autologous-derived exosomes elicit a negligible immune response or an inflammatory reaction, as well as possess the ability of self-degradation and active/passive targeting *via* surface or gene modification. These characteristics make exosomes more suitable than other carriers to improve the biocompatibility of chemical drugs (120). A study developed A33 antibody, which is uniformly expressed in colorectal cancer, positive exosomes loading doxorubicin (Dox) to improve the targeting ability, and this system showed high efficacy for the treatment of colorectal cancer without causing cardiotoxicity. Additionally, it demonstrated that exosomes fused with target cells more efficiently under acidic conditions, implying that exosomes facilitated drug release in acidic cancer cells (**Figure 4A**) (112). Furthermore, autologous exosomes can also reduce the hepatotoxicity and nephrotoxicity of gemcitabine by mediating the delivery of drugs to the cancer site due to its special homing effect (117). Additionally, the use of exosomes increased cancer cell uptake, minimized immunogenicity, and enhanced the biocompatibility of aspirin (**Figure 4B**) (115).

### Application in Overcoming Multidrug Resistance

Clinically, multidrug resistance is a fatal blow to cancer chemotherapy. Additionally, the TME is well known for its potential for drug resistance. In fact, exosomes play a crucial role in this process since they transmit the information related to drug resistance between cancer cells. On the contrary, for cancer treatment, exosomes can also be used to transmit information about reversing drug resistance between cancer cells. Thus, caudally injected exo-anti-miR-214 was shown to reverse the resistance to cisplatin in gastric cancer and repress cancer growth *in vivo* by downregulating the expression of miR-214, which promoted gastric cancer chemoresistance (121). Additionally, in pancreatic ductal adenocarcinoma, chemoresistance to gemcitabine (GEM) significantly reduced the sensitivity of chemotherapeutic agents. The downregulation of key proteins, such as human equilibrium nucleotide transporter 1 (hENT1) and deoxynivalenol kinase (dCK), reduced the transport of GEM to the cytoplasm and insufficient conversion of GEM phosphorylation products, resulting in GEM-related chemoresistance. The internalization of exosomes into the cell was shown to be energy-dependent and was mediated by clathrin-independent endocytosis and macropinocytosis. Thus, GEM could enter the cytoplasm through a different internalization *via* exosomes, avoiding the primitive transport involved in human equilibrium nucleotide transporter protein 1 (hENT1) and thus, resist resistance (116). Drug resistance has been associated with an efflux transporter known as P-glycoprotein (P-gp) that is located in the cellular membrane (122). A study showed that the PTX-loaded exosome could bypass P-gp-mediated drug efflux in the resistant cancer cells to overcome the chemoresistance (**Figure 4C**) (123).

### Application in Brain Cancer Chemotherapy

Due to chemoresistance and the limitations related to BBB, most chemotherapy drugs show poor therapeutic efficacy in brain tumors. The exosome is an extraordinary carrier with improved drug bioavailability, biocompatibility, and the ability to cross the BBB, which is helpful in improving the efficacy of drugs. For example, doxorubicin- and paclitaxel-loaded exosomes can cross BBB smoothly, with good bioavailability and anticancer efficacy (**Figure 4D**) (124). In another study, neuropilin-1-targeted peptide (RGE) was conjugated with the exosome membrane to improve its biocompatibility (110). As previously mentioned, brain cancer is also affected by multidrug resistance. It has been proved that miR-9 plays an important role in the development of drug resistance in glioblastoma cells against temozolomide by inducing an increase in P-gp. After entering the cells by exosomes, anti-miR-9 could reverse the expression of the multidrug transporter and sensitize glioblastoma cells to temozolomide (125). Thus, exosomes can be used to increase the therapeutic efficacy in brain cancer, especially in crossing BBB and overcoming chemoresistance.

In summary, the anti-tumor effect of chemotherapy was mainly inhibited by the low bioavailability and poor biocompatibility of the drug and the drug resistance in cancer cells. Exosomes could improve their bioavailability, stability, trans-biological barrier capacity, and overcome tumor drug resistance, improving the antitumor effect of chemotherapy.

## Use of Exosome in Combination With Cancer Immunotherapy

The immunosuppressive TME is known to help the cancer cells evade the immune system (126). For example, apoptotic tumor cells are rarely recognized as antigens, and TME has the ability to upregulate the expression of regulatory T (Treg) cells or myeloid-derived suppressor cells and downregulate that of the cytotoxic T cells (127). Therefore, the inhibition of immune escape is the focus of tumor immunotherapy (128). Recent studies have shown that tumor-derived exosomes (TEXs) and immune cell-derived exosomes (IEXs) exhibit outstanding immunomodulatory effects and can overcome tumor immunosuppression (**Table 4**) (143, 144). Among them, dendritic cell-derived exosomes (DEXs) and tumor cell-derived exosomes are widely used because of their high specificity (145).

**Table 4:** Exosome-assisted immunotherapy.

Source of exosomes	Tumor	Size	Characteristics	Ref
Ovalbumin (OVA)-pulsed dendritic cells	/	153 nm	1. Exosomes carry high levels of OVA and induce a more potent antigen-specific response. 2. Induce antigen-specific CD8+ T cells. 3. Induce antigen-specific IgG production. 4. Facilitate interferon gamma production in mouse splenocytes.	(129)
Natural killer (NK) cells exposed to the NB cell	Neuroblastoma	less than 100 nm	1. The exosomes secreted by NK cells exposed to NB cells showed efficient cytotoxicity against NB tumors. 2. The exosomes, capable of transferring their content into naive NKs, exerts a powerful cytotoxic effect on target cells. 3. Overcome the immune resistance of malignant cells.	(130)
HEK293T cells transfected with plasmid DNA encoding SIRPa variant	Colon adenocarcinoma	100 nm	1. Tumor-derived exosomes enhance the effectiveness of CD47-targeted therapy. 2. Tumor-derived exosomes induce innate and adaptive anti-tumor responses.	(131)
Glioblastoma cells	Glioblastoma	30 - 50 nm	1. Exosomes derived from tumor cells are effective antigens, which are convenient to store and extract, and can be used to load DCs. 2. DC loaded with exosomes presents highly efficient uptake, prolonged storage presentation and long-lasting processing. 3. Exosomes efficiently transfer antigens from professional antigen-presenting cells (APCs) to other APCs, facilitating antigen-specific immune responses.	(132)
Dendritic cell	Hepatocellular carcinoma	50-150 nm	1. DEXAFP elicited a strong antigen-specific immune response. 2. The tumor microenvironment was significantly improved following DEXAFP treatment, resulting an increase in CD8+ T lymphocytes and a decrease in regulatory T (Treg) cells.	(133)
Pancreatic cancer cells	Pancreatic cancer	/	1. TEX carries tumor-associated antigens. 2. Because TEX are preferentially recruited to the MHCII loading compartment it is rarely degraded by lysosomes 3. TEX is enriched in newly delivered MHCII molecules and preferentially activates CD4 helper T cells.	(134)
Dendritic cells	Cervical cancer	/	1. Dendritic cell (DC)-derived exosomes (Dexo) are capable of inducing anti-tumor immune responses. 2. Dexo effectively induced cytotoxic activity of CD8+ T cells against TC-1 tumor cells. 3. Dexo promoted the immune response to *in vitro* restimulation induced by antigen E7 in inoculated mouse splenocytes.	(135)
Hepatocellular carcinoma tumor cells	Hepatocellular carcinoma	30-100 nm	1. After DC-TEX treatment, the number of T regulatory cells decreased and the number of CD8+ T cells increased. 2. After DC-TEX treatment, the number of PD-1+ increased significantly.	(136)
Cell- and serum-derived exosomes	/	~140 nm	Fetal bovine serum-derived exosomes (bo-EXO), which have the ability to reach the surface zone (macrophage zone) and the paracortical zone (T-cell zone) of lymph nodes, are effective in delivering immunostimulatory molecules to antigen-presenting cells and T cells.	(137)
Natural Killer cells	Glioblastoma	~100 nm	1. Natural killer (NK)-exosomes have antitumor activity against glioblast cells. 2. Natural killer (NK)-exosomes have the ability of tumor targeting. 3. Natural killer (NK)-exosomes can be loaded with other anticancer drugs, enhancing their antitumor effects and tumor specificity and facilitating their passage through the BBB.	(138)
RenCa cells	Renal cell carcinoma	30-100 nm	1. Exosomes carry high levels of OVA and induce a more potent antigen-specific response. 2. Induces antigen-specific CD8+ T cells. 3. Induces antigen-specific IgG production. 4. Facilitate interferon gamma production in mouse splenocytes.	(139)
Fetal bovine serum	Breast cancer	109 nm	1. The exosomes secreted by NK cells exposed to NB cells showed efficient cytotoxicity against NB tumors. 2. The exosomes, capable of transferring their content into naive NKs, exerts a powerful cytotoxic effect on target cells. 3. Overcome the immune resistance of malignant cells.	(140)
Tumor cell	/	/	1. Tumor-derived exosomes enhance the effectiveness of CD47-targeted therapy. 2. Tumor-derived exosomes induce innate and adaptive anti-tumor responses.	(141)
Bone marrow mesenchymal stem cell	Pancreatic ductal adenocarcinoma	140 ± 37.6 nm	1. BM-MSC-derived exosomes significantly improve tumor targeting and increase drug accumulation at the tumor site. 2. Protect the cargo gene.	(142)

“/” represents that information was not mentioned in the original article.

### Application in Immunotherapy of TEXs and IEXs

Tumor-derived exosomes are known to carry immunosuppressive signals from tumor cells and transmit them between cells (146, 147). However, they are rich in tumor antigens as they carry tumor cell-related information (134). Therefore, isolating TEXs and injecting them into the organism could help with antigen presentation, which could generate an immune response, and produce enough cytotoxic T cells to kill tumor cells.

**Figure 5:**
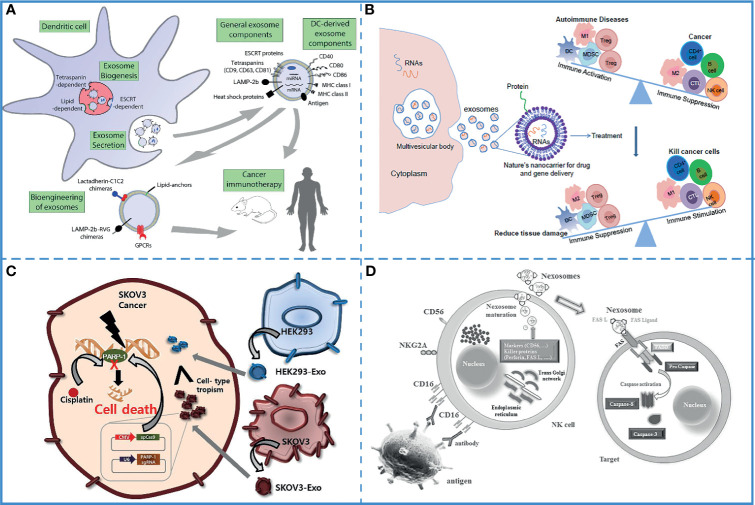
Immunotherapy. **(A)** DEXs can overcome tumor immunosuppression. Reproduced with permission from ref (150). Copyright 2015 Elsevier. **(B)** DC-derived exosomes have been used for immunotherapy in animal studies and human clinical trials. It can also be bioengineered to contain specific cargoes. Reproduced with permission from ref (155). Copyright 2014 Elsevier. **(C)** Compared with epithelial cell-derived exosomes (HEK293-exo), tumor -derived exosomes (SKOV3-Exo) accelerates their accumulation in SKOV3 tumor cells due to their cell tropism. Reproduced with permission from ref (156). Copyright 2017 Elsevier. **(D)** Nexosomes (NK-derived exosomes) entrap CD markers, perforin, and FAS-L, which mediates cytotoxicity by interacting with FAS on exposed cell membranes. Reproduced with permission from ref (130). Copyright 2017 Wolters Kluwer Health, Inc.

Several experiments have shown that the immunostimulatory ability of dendritic cell-derived exosomes (DEXs) is the same as that of the donor cells (148), and they directly or indirectly stimulate cytotoxic T cells *via* the major histocompatibility complex class I (MHC I) and major histocompatibility complex class II (MHC II) (149). Additionally, DCs are highly immunogenic, and different mature DC-derived exosomes have different functions. Mature, activated DC-derived exosomes often express MHC-I and MHC-II molecules as well as co-stimulatory molecules (CD40, CD80, CD86, etc.) and are therefore able to induce effective antigen-specific T and B cell responses and activate cytotoxic T cells and natural killer (NK) cells. Studies have shown that DC-derived exosomes induce antigen-specific responses against tumor cells by activating innate and adaptive immune cells and overcoming tumor-induced immunosuppression (**Figure 5A**) (150). Based on these characteristics, DEXs have been extensively used in free-cell tumor vaccines (133). Along with dendritic cells, the exosomes derived from macrophages, natural killer cells, B cells, and T cells also possess the immunoregulatory ability and can be used in tumor immunotherapy (151, 152).

### Application in Tumor Antigens or Adjuvants Delivering by TEXs and IEXs

The successful delivery of tumor antigens or adjuvants to antigen-presenting cells (APCs) is the most effective method to induce an anti-tumor immune response. Therefore, a novel carrier that can deliver tumor antigens and adjuvants to APCs is required for the development of cancer immunotherapy. Exosomes are natural vesicles produced by cells, which carry cargo molecules stably and safely, can smoothly cross biological barriers, and can target cells accurately. Furthermore, exosomes are also suitable as carriers in immunotherapy because (i) they can simulate the transmission of pathogens to antigen-presenting cells (153), (ii) they can carry a wide range of cargo molecules, such as tumor-specific antigens, peptides, and toll-like receptor (TLR) ligands (154), (iii) the loading method is optional and convenient, the cargos can be loaded directly into the exosomes isolated from the donor cells or indirectly by co-incubation with donor cells (**Figure 5B**) (155).

#### Application in Tumor Antigens or Adjuvants Delivering by TEXs

Compared with other exosomes, TEXs carry a higher number of tumor-associated antigens and can effectively target tumor cells (156) (**Figure 5C**). Additionally, the surface of TEXs is rich in proteins that promote cell-to-cell interaction; thus, they can be easily absorbed by the target cells (157). A study demonstrated that TEXs painted with the functional domain of high-mobility group nucleosome-binding protein 1 (TEX-N1ND), a potent adjuvant, enhanced DC immunogenicity; thus, eliciting long-lasting anti-tumor immunity and tumor suppression. Also, N1ND-decorated TEXs are known to promote N1ND uptake because of the surface proteins of TEXs (141). Another experiment used the early secretory antigenic target-6 (ESAT-6) from *Mycobacterium tuberculosis* as an immune response-inducing antigen and found that TEXs expressing ESAT-6 or its epitopes on the surface could be captured by APCs to induce immune responses against the tumor cells. The pathogenic antigen presented on the TEXs surfaces acted as an “artificial neoepitope” and was recognized by DCs as a “danger signal”, leading to the activation of an antitumor immune response (158). Furthermore, the TEXs-based tumor antigens-adjuvant co-delivery system was more efficient, which effectively activated DC2.4 cells and enhanced tumor antigen presentation capacity (159).

#### Application in Tumor Antigens or Adjuvants Delivering by DEXs

Compared with tumor cell lysates, DEXs are more easily absorbed by DCs and have excellent antigen storage, presentation, and processing ability (149). Meanwhile, compared with other microvesicles, DEXs can induce a more effective immune response. For instance, among the microvesicles and exosomes from antigen Ovalbumin (OVA)-pulsed DCs, only DEXs elicit antigen-specific CD8+ T-cells and antigen-specific IgG production (129). Another experiment demonstrated that DEXs loaded OVA with poly(I:C), a ligand of TLR-3, induced a strong protective immune response and markedly inhibited the tumor growth and improved the survival rate of the tumor-bearing mice (135). Similarly, thymic stromal lymphopoietin (TSLP) induced DEXs, which were enriched with OX40 ligand (OX40L), promoted proliferation of CD4^+^ T cells, upregulated the level of IL-4, and promoted Th2 differentiation (160).

#### Application in Tumor Antigens or Adjuvants Delivering by Other IEXs

The mutual communication between innate immune cells and acquired immune cells results in a perfect immune system, and the exosomes secreted by them are the bridge of their communication (152). For example, the exosomes secreted by innate immune cells can not only be received by other types of innate immune cells but also recognized by acquired immune cells, affecting the differentiation, activation, tissue recruitment, and production of cytokines by the acquired immune cells (**Figure 5D**) (124, 130). Therefore, exosomes derived from immune cells loaded with tumor antigens or adjuvants are easier to be recognized by the immune system; thus, activating the immune response and effectively killing the tumor cells (143). By carrying the adjuvant heat shock protein 70, these exosomes can effectively induce an immune response and cause tumor regression in the animal model (161). Additionally, T cell-derived exosomes loaded with CD40L can promote B cell proliferation and differentiation (162).

#### Different Application Between TEXs and IEXs Delivering Tumor Antigens or Adjuvants

Exosomes derived from immune cells and tumor cells loaded with tumor antigens or adjuvants are easily recognized by the immune system; thus, these are used as antigen carriers for cancer immunotherapy. Compared with IEXs, TEXs have a stronger ability to target tumor cells and efficiently deliver multiple tumor antigens to DCs (163). TEXs, which are recovered and enriched from patient serum might provide an optimized, individual-specific source of antigen for DCs vaccination (164). Nevertheless, numerous studies have shown that TEXs have immunosuppressive effects on the immune system since they can interfere with the maturation of DCs, weaken the activation of NK cells, induce suppressor cells of myeloid origin, and transform macrophages into tumor-promoting phenotype (165–167). Compared with TEXs, IEXs are more easily absorbed by DCs and have better antigen storage, presentation, and processing capabilities; hence, the exosome fraction is known to be more immunogenic *in vivo* and produces fewer side effects (168). However, treatment with single IEXs might be more resistant to immunomodulatory events occurring in tumors than other immunotherapies (149). IEXs can be modified by multiple methods to increase their targeting ability and become a promising delivery system (169, 170). Further research is required to fully utilize the advantages of TEXs and IEXs, and bypass their disadvantages to regulate tumor immunity, which has great application potential in cancer treatment (171).

### Application in Brain Cancer Immunotherapy of TEXs and IEXs

Based on their carrier and immunomodulatory ability, exosomes play a vital role in immunotherapy against the brain tumor. For example, co-delivery of tumor-derived exosomes with α-galactosylceramide (α-GalCer) on a DC-based vaccine induced strong activation and proliferation of tumor-specific cytotoxic T lymphocytes and destroyed tumor immunosuppressive environment, showing a powerful effect in glioblastoma (132). Additionally, DEXs loaded with chaperone-rich cell lysates (CRCLs) derived from GL261 glioma cells promoted proliferation and activity of CD4^+^ and CD8^+^ T cells, enhanced T cell infiltration in intracranial glioma tissues, and induced the generation of anti-tumor cytokines, including IL-2 and IFN-γ. Therefore, they significantly prolonged the survival of mice with tumors and inhibited tumor growth *in vivo* (172). A study used natural killer cell-derived exosomes (NK-Exos) to treat glioblastoma and found that NK-Exos exerted an innate therapeutic effect in glioblastoma. It demonstrated that NK-Exos possessed strong antitumor activity toward glioblastoma cells and could facilitate the transfer of other anti-cancer agents through the BBB by wrapping them (138).

In summary, collecting tumor-derived exosomes and reinjecting them into the organism could aid antigen presentation and elicit an immune response. While the dendritic cell-derived exosomes have the same immunostimulatory capacity as donor cells, the tumor-derived exosomes are able to activate innate and adaptive immune cells and overcome tumor-induced immunosuppression, triggering an antigen-presentation response against the tumor cells. Furthermore, in addition to their own biological activity, exosomes can transport tumor antigens or adjuvants to antigen-presenting cells and induce antitumor immune responses.

## Application of Exosome to Improve Cancer Photodynamic Therapy or Photothermal Therapy

Recently, substantial breakthroughs have been made in the use of near-infrared light (NIR) in anti-cancer treatment. However, both photodynamic therapy (PDT) and photothermal therapy (PTT) need an effective carrier delivery system to deliver photosensitizers (173) or photothermal transducers (174). PDT kills the cancer cells by irradiating the photosensitizer to produce the toxic singlet oxygen. On the contrary, PTT induces the thermal killing effect by using photothermal transducers to convert light energy into thermal energy. Currently, exosomes are being used as carriers in PDT and PTT to improve their effectiveness.

### Application in Photodynamic Therapy

**Figure 6:**
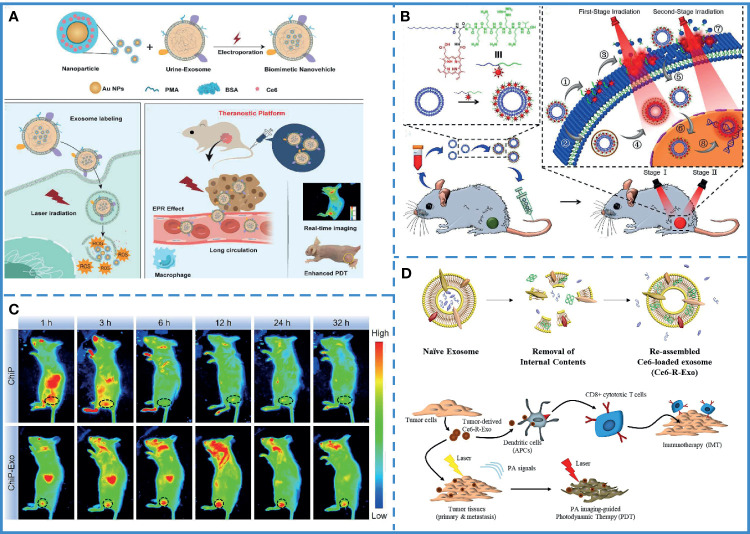
Photodynamic therapy. **(A)** Synthesis of Exo-PMA/Au-BSA@Ce6 nanovehicles. Laser irradiation at 633 nm under acidic conditions resulted in the collapse of the structure of nanovehicles, leading to the release of PMA/Au-BSA@Ce6 nanoparticles into the cancer cells, producing singlet oxygen species, inhibiting tumor cell growth. Reproduced with permission from ref (63). Copyright 2019 Elsevier. **(B)** Intravenous injection of ChiP-Exo and dual-stage light-guided PDT against the tumor. ChiP-Exo underwent the following steps: (1) PM-targeted delivery; (2) Endocytic delivery; (3) PM fluctuation; (4) Lysosomal escape after the first-stage light irradiation; (5) Photochemical internalization (PCI); (6) Nucleus-targeted translocation; this was followed by (7) PM rupture, and (8) Nucleus destruction under the second-stage light irradiation. Reproduced with permission from ref (175). Copyright 2019 Elsevier. **(C)** ChiP has several limitations, including a relatively shorter half-life *in vivo* and poor retention in tumor tissues. On the contrary, ChiP-Exo continuously accumulates in the tumor and exhibits superior tumor treatment ability. Reproduced with permission from ref (175). Copyright 2019 Elsevier. **(D)** Combined photodynamic therapy and immunotherapy *via* exosomes to treat tumors. Reproduced with permission from ref (176). Copyright 2020 Elsevier.

Currently, the transport efficiency of photosensitizers in PDT is low. Recent studies on the excellent carrier performance of exosomes have attracted the attention of researchers. Studies have shown that exosomes can smoothly carry the photosensitizer to the tumor site. The engineered exosome can be endowed with the ability to target tumor cells; thus, enhancing the therapeutic efficiency of PDT. For example, exosomes collected and purified from the urine of gastric cancer patients have the ability to passively target tumor sites due to natural cell membranes and antigens. Meanwhile, the exosomes are known to promote the internalization of Exo-PMA/Au-BSA@Ce6. Au NPs are transferred into aqueous solution by amphiphilic polymer (PMA) coating, and after surface coupling of the BSA layer, Ce6 is trapped into the BSA network and finally embedded in the exosome nanocarriers toward the cancer cells, avoiding endocytosis by macrophages and prolonging their blood circulation time. The results have shown that the nanoparticles could be released inside cancer cells under 633 nm laser irradiation, producing a large number of singlet oxygen species, killing the tumor cells (**Figure 6A**) (63). However, the short lifespan and limited diffusion length of cytotoxic reactive oxygen species (ROS) generated by the photosensitizer reduces the anti-tumor effects. Therefore, a nucleus targeted exosome was designed to enhance the PDT effect, engineered using a chimeric peptide (ChiP-Exo). In this study, a dual-stage light strategy was used for precise PDT by sequentially destroying the PM and nucleus of the cancer cells. Based on these exosomes, it could activate ROS *in situ* to disrupt the nuclei, which greatly inhibited tumor growth with minimized systemic toxicity (**Figures 6B, C**) (175). As mention above, tumor-derived exosomes can be used as immunostimulants. Based on this feature, the combination of photodynamic therapy and immunotherapy can be used to treat tumors through exosomes. For instance, recombinant exosomes derived from tumors (R-Exo) are used as drug delivery vehicles and immunostimulants at the same time. During exosomal re-assemble, a chlorin e6 photosensitizer was loaded into tumor-derived exosomes. After this modification, R-Exo retains its original average size and has the same membrane protein, so that it can target tumor cells. The R-Exo loaded with chlorine e6 (Ce6-R-Exo) can be observed by photoacoustic imaging, and it can effectively produce reactive oxygen species inside tumor cells under laser irradiation. In addition, Ce6-R-Exo enhances the ability of immune cells to release cytokines, which indicates that these modified exosomes can also be used as immunotherapeutic (**Figure 6D**) (176)

### Application in Photothermal Therapy

**Figure 7:**
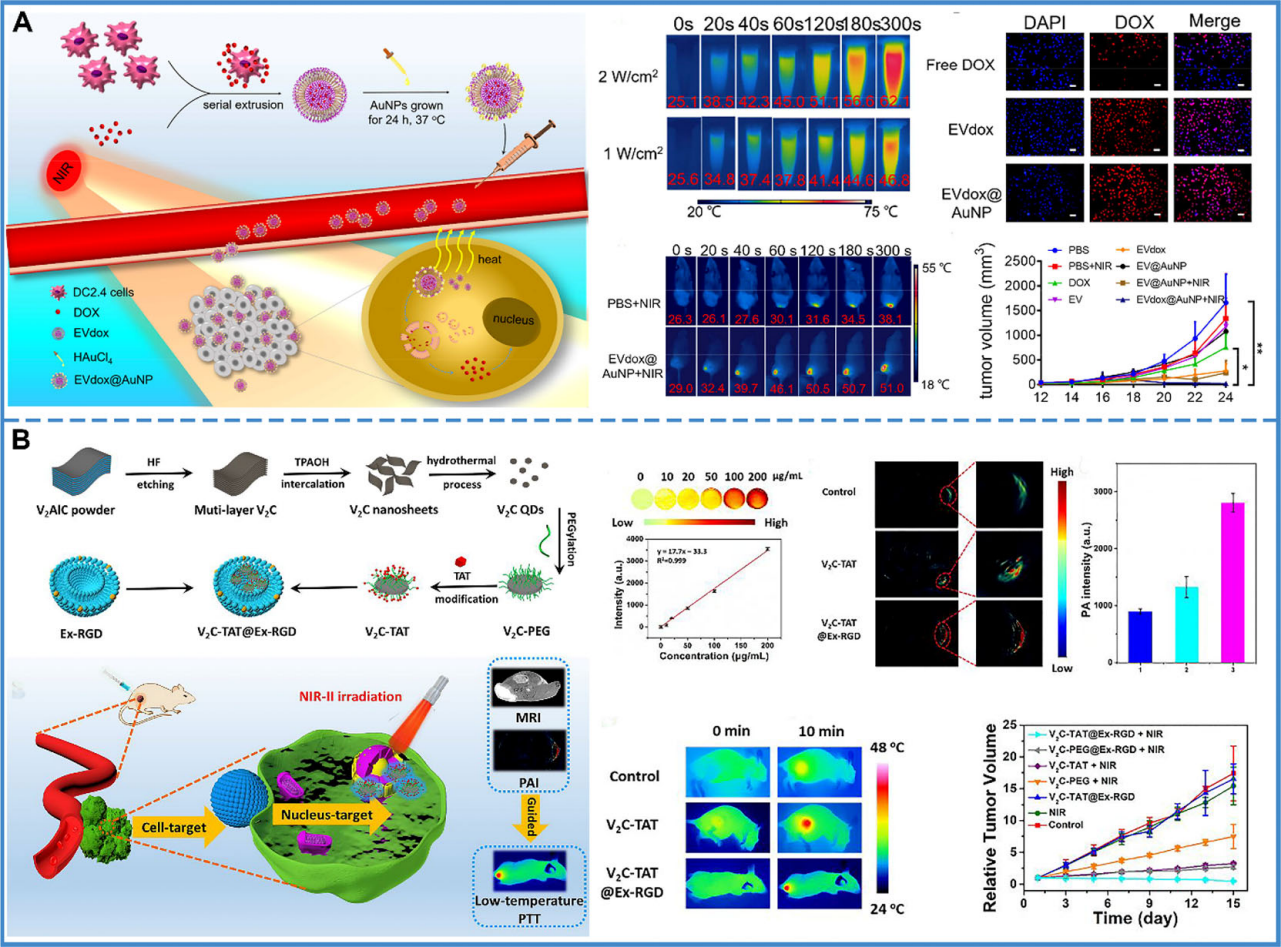
Photothermal therapy. **(A)** The prepared EVdox@AuNP (EVs encapsulating DOX were first obtained (EVdox), and self-growth of gold nanoparticles around EVdox) plays a synergistic role of photothermal and chemotherapy for tumor treatment. In this system, AuNPs have a good thermal effect and exosomes can assist DOX aggregation within tumor cells, thus inducing effective tumor elimination. Reproduced with permission from ref (177). Copyright 2019 Elsevier. **(B)** V2C-TAT@Ex-RGD (The small fluorescent V2C QDs were modified with TAT peptides and packaged into Ex with RGD modification) targeted the cancer cell membrane & nucleus organelle for low-temperature imaging-guided PTT in NIR-II. The PA intensity at the tumor site was 2.11-fold higher in V2C-TAT@Ex-RGD-treated mice than in V2C-PEG-TAT-treated mice. It was shown that the efficient tumor accumulation of V2C-TAT@Ex-RGD was due to the Ex-RGD coating. After 10 min of 1064 nm laser irradiation at a power density of 0.96 W/cm^2^, the tumor site warming was higher in the V2C-TAT@Ex-RGD-treated mice compared with the control group and the V2C-TAT-treated mice. And, the V2C-TAT@Ex-RGD +1064 nm laser irradiation group could effectively inhibit tumor growth without recurrence. Reproduced with permission from ref (178). Copyright 2019 American Chemical Society.

Compared with traditional therapy, PTT is controllable, noninvasive, and has minimal adverse effects. With the help of exosome delivery, photothermal transducers are carried to tumor cells and produce a thermal effect after NIR irradiation. For instance, an exosome with gold nanoparticles has been designed that assembles into a popcorn-like nanostructure. The formulated nanopopcorn, consisting of self-grown gold nanoparticles and exosomes loaded with DOX, was shown to retain the photothermal conversion of gold nanoparticles and cytotoxicity of DOX. The use of exosomes improved cellular internalization, controlled drug release, enhanced anti-tumor efficacy, and minimized adverse effects (**Figure 7A**) (177). However, it was found that PTT was blocked by heat shock proteins and other stress proteins, resulting in thermal tolerance. Thus, a higher temperature was required to achieve anti-tumor effects. However, the high temperature could damage the surrounding tissue. Therefore, the low-temperature nucleus-targeted PTT in the NIR-II region is conceptualized. For example, vanadium carbide quantum dots (V2C QDs) with good photothermal effect in the NIR-II region were modified with TAT peptides and packaged into exosomes with RGD modification. This carrier exhibited good biocompatibility, long circulation time, and endosomal escape ability. More importantly, it could target the cell and enter into the nucleus to achieve low-temperature PTT with advanced tumor destruction efficiency (**Figure 7B**) (178).

In summary, both PDT and PTT require delivery of the photosensitizer to the treatment site. Exosomes, as carriers of the photosensitizer, can increase the circulation time of the photosensitizer in the blood, and help the photosensitizer cross the blood-brain barrier. Additionally, the modified exosomes could carry photosensitizers to target the nucleus and improve the effectiveness of PDT or PTT.

## Patents of Exosomes in Cancer Therapy

In the past ten years, research on exosomes has grown exponentially. The functions of exosomes have expanded from transporting intracellular garbage to participating in cell-to-cell communication as well as tumor growth and metastasis. Despite this growing trend, the lack of reference standards for exosomes, the inability to replicate experimental results, and the lack of clinical transformation have resulted in an incomplete acceptance of exosomes in some scientific fields. Fortunately, many patents are trying to address these problems, and many more are expected to support the multiple roles of exosomes.

Currently, there are several techniques to separate exosomes with their individual advantages and disadvantages; however, there is no uniform standard of characterization for isolated exosomes, which adds uncertainty to further experiments. In response, EP3690434 provided a method to analyze EVs using the size-specific fractionation ability of size-exclusion chromatography and specific binding of probes to EVs. This method could speed up and facilitate the quantitative analysis of EVs (types and quantities) present in the samples, the physicochemical analysis of EVs, and an analysis of the specificity or affinity of binding of the probes to the components of EVs. This kit could detect whether the isolated exosomes contained other vesicles, which can be used as a standard for testing the purity of exosomes. In view of the low output of exosomes, researchers have proposed to artificially synthesize exosome analogs with identical physiological functions and can be mass produced.

Tumor-derived exosomes contain tumor markers and can be used in tumor diagnosis. If the clinical transformation of tumor diagnosis by exosomes can be achieved, the early detection of tumors and timely treatment will be possible and would significantly benefit the patients. Currently, several kits have been developed for the detection of different tumors and applied for patents. WO2020159181 developed a kit to predict the recurrence probability of acute myeloid leukemia by detecting CD53 and CD47 expression of exosomes in the blood collected from the patients; WO2020147252 developed a kit to detect breast cancer, mainly to detect the TII protein of exosomes derived from the body fluids of the patients; WO2019093717 developed a kit to diagnose lung cancer and predict prognosis. The use of exosomes in tumor diagnosis has substantial future prospects. The nearly noninvasive, simple, and rapid diagnosis technique can eliminate the concerns of patients and improve the rate of early diagnosis of tumors.

Many experiments have confirmed that exosomes can significantly improve the efficacy of tumor treatment, but most of them are in the experimental stage and need to gradually transition to clinical applications. Thus, it is necessary to apply for patents to promote the transformation of exosomes into clinical practice. Exosomes as carriers can increase the efficacy of drug therapy, but most of the synthesis techniques are difficult to reproduce, which is not conducive to further research. WO2020132946 and WO2020141369 have provided a detailed process of preparing exosomes to carry cargo molecules for tumor treatment and have recorded the conditions, reagents, and instruments used in the preparation process, with high reproducibility, which will be implemented in clinical trials in the future. Additionally, the addition of the function of exosomes as a carrier to the original research would help with the further advancement of technology and would help maintain the patent protection period. Apart from being a carrier, exosomes themselves have an anti-tumor effect; WO2020032379 confirmed that exosomes derived from macrophages exposed to death cells could reach cancer cells and inhibit the epithelial-mesenchymal cell conversion in cancer cells as well as infiltration of cancer cells; thereby preventing or treating cancer. The successful application for the patent of exosomes for tumor therapy indicates that exosomes have great potential in assisting chemotherapy, RNA interference therapy, immunotherapy, and phototherapy (**Table 5**), which can significantly improve the efficacy of tumor treatment and optimize the current tumor treatment strategy.

**Table 5:** Patents of exosomes.

Patent/Application Number	Title	Description	Tumor	Source	Therapeutic strategy	Country	Filling Year
EP3548005	Exosomes for delivery of therapeutic agents	This invention provides a method of preparing an exosome as drug delivery vehicles, and a method of making a composition which a therapeutic agent encapsulated within such an exosome, as well as methods of delivering such exosome and composition to specific tissues and organs.	/	Milk	Chemotherapy	European	2017
CN106943432	Umbilical cord mesenchymal stem cells-derived exosome and application of exosome in preparation of medicine for treating liver cancer	This invention focuses on the preparation of umbilical cord mesenchymal stem cell-derived exosome-loaded compound CP1-6, which has a significant inhibitory doing effect on hepatocellular carcinoma.	Liver cancer	Umbilical cord mesenchymal stem cells	Chemotherapy	China	2017
WO2017223186	Exosome-guided treatment of cancer	This invention is a novel assay for detecting the efficacy of cancer treatment by analyzing proteins in exosomes associated with mutations known to drive growth, metastasis, and/or proliferation.	Extensive treatment	Whole blood, serum, plasma, and urine	Immunotherapy/chemotherapy/RNA interference therapy	USA	2017
CN107119015	Exosome as well as preparation method and application thereof in preparing medicine for treating lung cancer	This invention provides a new preparation method that enables efficient *in vitro* exosome preparation by stimulating the cellular secretion of exosomes using KRN7000 and ATP, and the prepared exosomes have relatively good cytotoxic effects and lung antitumor activity.	Lung cancer	Umbilical cord blood cell	Immunotherapy	China	2017
CN109432427	Preparation method of tumor targeting heat therapy material taking exosome as carrier and product of preparation method	This invention provides a method of preparing a thermal therapy material with tumor-targeting ability using exosomes as carriers, the composition having photothermal efficacy and targeting lung cancer.	Lung cancer	Lung cancer cell A549	Phototherapy	China	2018
US20180193266	Exosomal compositions and methods for the treatment of disease	This invention provides an exosome composition that treats cancer by intranasal, intraoral or intratumoral administration	Extensive treatment	Bodily fluid	Chemotherapy	USA	2018
WO2019128952	Method for preparing immune cell exosome carrying chimeric antigen receptor and application thereof	The invention focuses on exosomes from chimeric antigen receptor (CAR) T cells in cancer therapy	Extensive treatment	T cell of patients	Immunotherapy	China	2019
CN110075122	Therapeutic exosome medicine for liver cancer	This invention provides an exosome with down-regulated VPS35 gene expression secreted by hepatocellular carcinoma cells, and mRNA molecule for the treatment of hepatocellular carcinoma.	Liver cancer	Liver cancer	RNA interference therapy	China	2019
WO2018151445	Exosome-based nanoparticle composite and method for preparing same	This invention provides a new exosome-based nanoparticle composite, and a method for its preparation. The nanocomposite improves *in vivo* stability and redispersibility through biocompatible polymers.	Breast cancer	RAW264.7 macrophage cell/breast cell line MDA-MB231	Chemotherapy	Korean	2019
CN110051833	Method for preparing cancer vaccine by using leukemia cell exosome	This invention is a cancer vaccine prepared using an exosome secreted by leukemia cells	Leukemia	Leukemia cell	Immunotherapy	China	2019
CN109675032	Medicament prepared from photothermal material and exosome-mediated chemotherapeutic drug and application thereof	This invention discloses a medicinal composition comprising a photothermal material and an exosome-mediated chemotherapeutic drug.	Breast cancer/liver cancer/cervical cancer	Breast cancer cell/liver cancer cell/cervical cancer cell	Phototherapy and chemotherapy	China	2019

Until January 2021, there were 205 clinical trials related to exosome research. Of the 205 trials, around 87 trials involved cancer-related studies (179). Among these clinical trials, exosome-based phase I and II clinical trials have been completed in advanced lung cancer patients showing promising data (148). Notably, DEX-based phase I and II clinical trials have been performed in non-small cell lung cancer (NSCLC), indicating the safety and feasibility of the approach, as well as the preference of these nanovesicles to stimulate T cell- and NK cell-based immune responses in patients (149). A vaccination trial with tumor antigen-loaded dendritic cell-derived exosomes showed the safety and feasibility of DEXs vaccines (NCT01159288). Based on these promising results of exosomes for clinical treatment, significant progress in this field could be expected in the future.

## Conclusions and Future Perspective

In 1983, exosomes were first discovered in sheep reticulocytes, were named “exosomes” by Johnstone in 1987. Since its discovery, exosomes have always been regarded as a way for cells to excrete waste. Now, with many studies on its biological origin, material composition and transport, intercellular signal transduction, and distribution in body fluids, it has been found that exosomes have several functions. All body fluids contain exosomes, such as blood, saliva, urine, cerebrospinal fluid, and milk. Although exosomes come from a wide range of sources, their yield is very small. There are many kinds of purification methods, the most common one being ultracentrifugation, which results in large amounts of exosomes, but with low purity, and some researchers have proposed that the exosomes may contain other EVs rather than being pure exosomes. Another method is sucrose-gradient centrifugation, which generates highly pure exosomes, but the process is complicated and has a low yield. Additionally, there are filtration centrifugation, immunomagnetic beads method, PS affinity method, chromatography, etc. It is important to note that since there are multiple methods of separation and purification, there is a wide gap in the characteristics of the isolated exosomes. Therefore, a reliable and unified standard is needed to purify, separate, and characterize the exosomes.

Exosomes have several advantages as drug delivery systems, such as good biocompatibility, low immunogenicity, targeting specificity, and the ability to overcome membrane barriers. Based on these characteristics, exosomes are widely used in tumor diagnosis, prognosis, treatment, and are gradually applied to clinical trials. For tumor diagnosis, TEXs carry tumor specificity markers, which can be detected by laboratory tests or imaging analysis, to achieve early detection and early treatment. Furthermore, by analyzing the composition of TEXs, we can evaluate the short-term therapeutic effect, which would act as a reference for clinical treatment. Apart from providing an efficient and safe drug delivery system, exosomes display different synergistic effects in different tumor treatment strategies to reduce the adverse effects and improve the therapeutic effect. For example, in RNAs interference-based therapy, exosomes can not only ensure the stability of RNA but also reduce the risk of off-target effects; for chemotherapy, exosomes can not only efficiently transport drugs but also minimize drug resistance; for immunotherapy, the role of exosomes is more diverse, TEXs can be used as tumor antigens without any modification, thus activating the immune system. Moreover, exosomes derived from different sources of immune cells can not only have the same immune function as donor cells but also modulate the TME and inhibit tumor immune escape. In PDT/PTT, the exosomes can transport more photosensitizers and photothermal transducers into tumor cells. Also, as the exosome inherits the heat sensitivity of the lipid membrane, it is easy for materials to achieve endosomal escape after light. Interestingly, exosomes purified from patients themselves can be used in personalized therapy.

Brain tumors are different from other types of tumors due to the special position of the brain and the tight barrier system. Brain tumors are not easy to diagnose and even more difficult to treat. Many general tumor treatment strategies are not effective for brain tumors, and thus, the fatality rate of brain tumors remains high. The high permeability, biosafety, and exosome targeting are indispensable factors in the brain tumor treatment strategy. Many experiments have shown that the addition of exosomes to the original tumor treatment strategy can greatly improve the therapeutic efficacy of brain tumor treatment. Exosomes are like a natural ferry, which can carry the necessary substances for treatment across the BBB without destroying its integrity. Thus, the addition of exosomes might promote the treatment of brain tumors and reduce their mortality.

## Author Contributions

YZ and TC contributed to the conception and design of the study. YZ and PL wrote the first draft of the manuscript. HT drew the drawings and collected the data. XC, QW, and TC revised the manuscript. All authors contributed to the article and approved the submitted version.

## Funding

Financial supports from the Guangdong Basic and Applied Basic Research Foundation (2019B1515120043), the Research Fund of University of Macau (File no. MYRG2019-00121-ICMS and MYRG2018-00207-ICMS), the Science and Technology Development Fund, Macau SAR (File no. 0098/2020/A), and the Key Project of Basic Research of Shenzhen (JCYJ20200109113603854) are gratefully acknowledged.

## Conflict of Interest

The authors declare that the research was conducted in the absence of any commercial or financial relationships that could be construed as a potential conflict of interest.

## Publisher’s Note

All claims expressed in this article are solely those of the authors and do not necessarily represent those of their affiliated organizations, or those of the publisher, the editors and the reviewers. Any product that may be evaluated in this article, or claim that may be made by its manufacturer, is not guaranteed or endorsed by the publisher.
